# Surgical strategies for treatment of malignant pancreatic tumors: extended, standard or local surgery?

**DOI:** 10.1186/1477-7819-6-123

**Published:** 2008-11-12

**Authors:** Matthias Glanemann, Baomin Shi, Feng Liang, Xiao-Gang Sun, Marcus Bahra, Dietmar Jacob, Ulf Neumann, Peter Neuhaus

**Affiliations:** 1 Department of General, Visceral, and Transplantation Surgery, Charité, Campus Virchow Klinikum, Universitätsmedizin Berlin, Germany; 2 Department of Hepatobiliary Surgery, Shandong Provincial Hospital, Clinical College of Shandong University, Jinan, PR China

## Abstract

Tumor related pancreatic surgery has progressed significantly during recent years. Pancreatoduodenectomy (PD) with lymphadenectomy, including vascular resection, still presents the optimal surgical procedure for carcinomas in the head of pancreas. For patients with small or low-grade malignant neoplasms, as well as small pancreatic metastases located in the mid-portion of pancreas, central pancreatectomy (CP) is emerging as a safe and effective option with a low risk of developing de-novo exocrine and/or endocrine insufficiency. Total pancreatectomy (TP) is not as risky as it was years ago and can nowadays safely be performed, but its indication is limited to locally extended tumors that cannot be removed by PD or distal pancreatectomy (DP) with tumor free surgical margins. Consequently, TP has not been adopted as a routine procedure by most surgeons. On the other hand, an aggressive attitude is required in case of advanced distal pancreatic tumors, provided that safe and experienced surgery is available. Due to the development of modern instruments, laparoscopic operations became more and more successful, even in malignant pancreatic diseases. This review summarizes the recent literature on the abovementioned topics.

## Background

Various pancreatic diseases demand surgery, among which malignant tumor resection is the mainstay of pancreatic surgery, including local, partial, or total pancreatectomy. Pancreatic tumor removal has during the recent years become a routine surgical procedure, and the resection rate of affected patients has markedly increased within the last decades. Consequently, much progress has been made and several consensuses on surgical principles have also been reached.

Indeed, pancreatic surgery may nowadays include additional venous and/or arterial vascular resection as well as defined lymphadenectomy or removal of adjacent organs in terms of multivisceral surgery along with resection of the main affected part of the organ. However, long-term survival has not increased in the same way as perioperative morbidity and mortality have decreased during this period. Therefore, the different surgical strategies, all once implemented to improve long-term outcome, need to be evaluated once more.

Since it is clear that an almost complete tumor removal instead of conservative treatment modalities is beneficial for the patient, we will focus in this review on surgical strategies of tumors which have infiltration the surrounding tissue or vessels. Thus, we report on extended pancreatic surgery, especially on the extent of lymphadenectomy, arterial and venous vessel resection, and total pancreatectomy. With regard to local tumor removal, like in centrally located neoplasms, central pancreatectomy (CP) has become useful with effective preservation of both cephalic and distal pancreatic remnants [1]. Moreover, the laparoscopic technique, one of the best examples of modern surgery, is now more widely used for the diagnosis and treatment of pancreatic tumors including laparoscopic distal pancreatomy and laparoscopic pancreatoduodenectomy [2].

In this article, we present an updated overview of the literature on the above topics and also their benefits for pancreatic surgeons.

## Extended pancreatoduodenectomy

Pancreatoduodenectomy (PD) has been regarded as the standard operation for pancreatic head carcinoma, and can be performed safely with a mortality of 0.7%–3% and morbidity of 36%–41% in high-volume centers [3,4]. Nonetheless, outcome is not encouraging, since the five-year patient survival is still less than 5%–20% [5]. From an analysis of 4005 patients who underwent resection for pancreatic adenocarcinoma, the overall median survival was 13 months, and 5-year survival was only 6.8% [6].

Thus, many surgeons attempted to improve survival via a more radical or "extended" operative technique. Extended pancreatectomy (EP) is a term that was used to describe several variations of the standard pancreatoduodenectomy or distal pancreatic resection. Initially it was proposed by Fortner et al. in the early 1970's as regional pancreatectomy, a method for more aggressive nodal or marginal clearance [7,8]. Nowadays EP is similar to pancreatectomy with extended lymphadenectomy and combined resections of adjacent vessels, retroperitoneal structures and organs. Reddy et al. recently defined it as a procedure which may include (a) total pancreatectomy (TP), (b) extended lymphnode dissection (ELND), and (c) portal/mesenteric vascular resections (VR) [3].

### Extended lymph node dissection (ELND)

As defined at an international congress in 1998 [9], the standard resection comprises of regional lymphadenectomy around the duodenum and pancreas, including the lymph nodes on the right side of the hepatoduodenal ligament, the right side of the superior mesenteric artery, and the anterior and posterior pancreaticoduodenal lymph nodes, in addition to the common (pylorus-preserving) PD operation. A lymphadenectomy outreaching the abovementioned area could therefore be considered an extended lymphadenectomy [5,9].

The rationale for PD with ELND is based on the high incidence of intra- and extrapancreatic neural invasion (65%) in pancreatic cancer, as well as the high incidence of lymphnode metastases (30–75%) [5,10,11]. In recent years however, surgical results of ELND showed improved 5-year survival rates compared to standard lymphadenectomy. Manabe and co-workers supported these findings with 5-year survival rates of 33.4% versus 0% for PD with ELND compared to PD only [12]. Pedrazzoli and colleagues randomized 81 patients with pancreatic cancer to either standard PD or PD+ELND, and showed that patients with positive lymph nodes had a significantly better survival rate after PD+ELND [13].

However, recent prospective studies did not show any significant differences in 5-year patient survival between ELND and standard lymphadenectomy (mortality less than 6,5%; morbidity above 36%) [3].

Therefore Michalski and his colleagues carried out a systematic review and meta-analysis to compare the survival rates following PD with and without extended lymphadenectomy. Of the 484 potential studies on lymphadenectomy in pancreatic cancer, 159 cases were with standard lymphadenectomy, while 160 were with ELND. Overall, no significant differences in survival were found, whereas morbidity tended to occur more often in the ELND group with diarrhea and delayed gastric emptying [14].

Doi et al. retrospectively studied 133 patients who underwent margin-negative PD with ELND. The result showed that 84% of patients who had positive para-aortic lymph nodes died within 1 year compared to 46% with negative lymph nodes. Moreover, their multivariate analysis revealed that lymphatic metastasis of para-aortic lymph nodes was a single independent factor for increased mortality. They concluded that in case of para-aortic nodal metastasis extended resection should not be considered [15]. This statement may be underlined by the recent results from the Mayo Clinic, in which they demonstrated in 104 patients with pancreatic carcinoma that the presence of regional nodal metastasis was associated with a poor survival (p = 0.006) [16].

Farnell et al. reviewed four prospective randomized trials comprising of 424 patients. These studies showed no benefit in long-term survival in the PD+ELND group even with comparable morbidity and mortality rates. 3-year and 5-year survival rate reached about 41% and 16% respectively. Nevertheless, postoperative diarrhea in the early months after surgery was more severe in patients undergoing ELND [17].

In conclusion, standard lymphadenectomy should be the procedure of choice in PD for pancreatic cancer, whereas ELND should only be performed within randomized controlled trials, if at all [3,14].

### Portal/mesenteric vein resection (VR)

Portal or superior mesenteric vein involvement with the tumor is very common in pancreatic carcinoma, because of its anatomical site and infiltrative characteristic. Therefore, vascular resection should be performed to achieve a negative resection margin. Moore and colleagues from the University of Minnesota already reported as early as 1951 on SMV resection and reconstruction [18]. The original objective for concomitant vascular resection was to increase the resectability and consequently improve the rate of R0 resections, however, the histological outcome was not as good as expected. Indeed, portal vein invasion was detected in almost 63% of 1.646 patients from 52 studies, but 40% of venous vessel resected patients still had a tumor positive margin (0–85%) [19]. In the same line of evidence, Tseng et al. reported on 291 patients who underwent PD with 110 of them requiring additional VR. In these, R1 resections were more common (22%) compared to standard PD (12%), but contrary to earlier reports, median survival was not negatively affected, reaching 23.4 versus 26.5 months, respectively (p = 0.177)[20,21].

Similarly, in a study comparing the survival of 22 patients who underwent PD with VR to 54 patients without VR, a slight survival benefit was noted in the patients without VR (33.5 versus 20% after 5 years, p = 0.18), however not reaching any statistical significance [16]. Similar results were reported by Riediger et al. who published a retrospective study with 53 of 222 (24%) patients requiring additional VR during PD. They observed that almost 60% of cases had true tumor involvement of the venous wall, whereas 40% had no proven tumor infiltration. Similar to previous reports, morbidity and mortality were comparable between patients with and without VR (morbidity: 23% vs. 35%, mortality: 3.8% vs. 4.1%, respectively) [22].

Additionally, a review by Siriwardana et al. on the same topic based on 52 studies, revealed that additional VR to PD did not add to morbidity or mortality (median morbidity rate: 42% (9–78%), mortality rate: 5.9% (0–33%)). Median survival of patients with VR+PD was 13 months, and 1-, 3- and 5-year survival rates were 50, 16, and 7%, respectively [19].

Yekebas et al. also showed similar in-hospital morbidity (39.7% vs. 40.3%) and mortality rates (4% vs. 3.7%) when comparing patients requiring PD with additional VR (136 patients) or without (449 patients), as well as similar median survival (15 months vs. 16 months, p = 0.86) and two-year survival probabilities (36% vs. 34%, p = 0.9). Their multivariate analysis identified nodal involvement (N1) and poor tumor grading (G3) as the only predictors of decreased survival, while evidence of vascular invasion had no adverse impact on survival [23].

Overall, additional resection of the portal and/or superior mesenteric vein does not influence the general morbidity or mortality rates during PD and could therefore successfully be performed in order to achieve tumor free margins (Table 1). Major venous involvement is no longer an absolute contraindication to pancreatic resection. This procedure might achieve a similar long-term survival, provided a tumor resection with tumor free surgical margins can be achieved.

**Table 1:** Reports of pancreatoduodenectomy (PD) with simultaneous venous vessel resection (VR).

Author (Year of publication)	Number of PD with VR	Mortality [%]	Morbidity [%]	Survival
Tseng[21] (2006)	110	2	21	23.4 months (median) ^#^
Siriwardana[19] (2006)°	1.646	5.9 ^#^	42 ^#^	13 months (median); 5-YS: 7% ^#^
Riediger[22] (2006)	53	3.8 ^#^	23 ^#^	5-YS: 15% ^#^
Al-Haddad[16] (2007)	22	0	NA	5-YS: 20% ^#^
Yekebas[23] (2008)	136	3.7 ^#^	40.3 ^#^	15 months (median); 2-YS: 34% ^#^

**Overall**	**1.967**	**3.1**	**31.6**	

^# ^not statistically significantly different compared to PD without VR

° systematic review

NA: not available

YS: Year survival

### Hepatic, celiac, superior mesenteric artery resection

In contrast to venous resections, experience with arterial resection during PD is limited [19,21,23-25] (Table 2). Visceral arteries that are commonly involved in carcinoma, including the mesenteric, celiac, or hepatic artery, are rarely resected [21]. In a systemic review of 1.646 patients from 52 studies, only 7.1% had adjacent arterial resection (common hepatic artery, SMA, and celiac axis were most common)[19]. Nakao et al. performed 15 arterial resections out of 200 cases of curative pancreatic surgeries with vascular resection. Postoperative mortality was higher in these patients (35.7%) compared to those without arterial resection (1.1%) and those with VR only (2.7%). Long-term survival was also low and almost similar to that of unresected patients (0%)[24].

**Table 2:** Reports of pancreatoduodenectomy (PD) with simultaneous arterial vessel resection.

Author (Year of publication)	Number of PD with arterial vessel re-section	Mortality [%]	Morbidity [%]	Survival
Settmacher[26] (2004)	3	0	NA	10 months (mean)
Tseng[21] (2006)	17	2.1	21	23.4 months (median) ^# ^*
Siriwardana[19] (2006)	117	5.9 ^#^	42 ^#^	13 months (median); 5-YS: 7% ^#^
Nakao[24] (2006)	15	35.7 ^#^	NA	NA
Yekebas[23] (2008)	13	3.7 ^#^	40.3 ^#^	15 months (median); 2-YS: 34% ^# ^*
Stitzenberg[25] (2008)	12	17	100	20 months (median)

**Overall**	**177**	**6**	**50.8**	

# result of patients with both venous and/or arterial vessel resection

* not statistically significantly different compared to PD without vascular resection

° systematic review

NA: not available

YS: Year survival

On the other hand, Yekebas et al. reported that patients with resection of the hepatic artery or superior mesenteric artery had a similar median survival (15 months) to those without arterial resection, without increasing morbidity or mortality [23].

Stitzenberg et al. also reported on 12 out of 252 patients with pancreatic cancer who underwent PD with resection of a tumor-involved hepatic artery and/or celiac artery. They showed that arterial resection resulted in a similar median survival time compared to their patients without arterial resection (20 vs. 21 months) [25]. Indeed, Settmacher et al. showed that vascular resection and reconstruction during PD is possible [26].

Different from venous resection, which has already reached acceptance, simultaneous arterial resection still remains a matter of dispute. Due to the lack of relevant clinical data it is difficult to draw any universal conclusions on this issue [3].

## Extended distal pancreatectomy

Malignant tumors of pancreatic body and tail have traditionally been considered as a disease with a dismal prognosis due to early tumor spreading to adjacent or distant organs without specific symptoms at time of diagnosis. Consequently, these tumors are associated with a lower resectability rate of only 10–12%, although surgical resection is the only viable chance for cure of this aggressive cancer [27-29].

Therefore, survival is expected to be markedly improved by extending the standard operation to an extended distal pancreatectomy (DP) including resection of regional lymph nodes, retroperitoneal structures, surrounding vessels, and adjacent organs (stomach, spleen, colon, adrenal gland, etc) [30,31].

In most of the available studies focusing on extended DP, the overall mortality was less than 1%. The median survival ranged from 16 to 33 months, while 5-year survival rates between 19 and 42% have been reported (Table 3), which are still better than those reported after extended PD [29,31-38].

**Table 3:** Reports on extended distal pancreatectomy (EDP)

Authors (Year of publication)	Number of patients with EDP	Mortality [%]	Morbidity [%]	Survival
Ozaki[32] (1996)	15	0	NA	5-YS: 29%
Mayumi[33] (1997)	6	0	NA	1-YS: 40%^#^; 3-YS: 20%^#^
Sasson[34] (2002)	37	1.7	38	16 months (median)*; 5-YS: 26%
Shoup[29] (2003)	22	0	NA	15.9 months (median)* ^#^; 5-YS: 22%,10-YS: 18%* ^#^
Gagandeep[35] (2006)	3	0	NA	1-YS: 100%
Shimada[36] (2006)	88	0	NA	22 months (median); 5-YS: 19%
Teh[37] (2007)	33	3	36	5-YS: 36% (endocrine tumors)
Hirano[31] (2007)	23	0	48	21 months (median); 5-YS: 42%
Mohebati[38] (2008)	41	0	24	32.7 months (median)

**Overall**	**268**	**0.5**	**36.5**	

* not statistically significantly different compared to standard distal pancreatectomy

^# ^statistically significantly different compared to unresected patients

NA: not available

YS: year survival

In control studies by Sasson et al. and Shoup et al. patient survival after extended DP (required resection of surrounding structures) and standard DP showed no significant difference (26 months vs. 16 months; p = 0.08) [29,34], indicating that by applying an extended DP a similar long-term survival can be achieved when compared to patients suffering from pancreatic cancer without adjacent organ infiltration, a circumstance which is normally considered as a poor indicator.

Furthermore, tumor infiltration of celiac artery, portal vein, or other adjacent organs is usually regarded as unresectable. Extended DP can however to some extent result in a better long-term survival for these patients. The report of Shoup et al. showed that median survival following tumor resection was 15.9 months compared to 5.8 months in patients who were not resected (p < 0.0001). Actual 5 and 10 year survival rates were 22% and 18% respectively, or 8% and 0% if no resection was attempted because of locally unresectable disease [29]. In an analysis by Shimada et al. of 88 patients with extended DP, which is the largest series reported on, he demonstrated that only lymph node involvement and the degree of histologic vein invasion were independent predictors of long-term survival [36].

Celiac axis infiltration and resection is the biggest obstacle in extended DP and was once considered as a contraindication for tumor removal. Since Appleby et al. first proposed en-bloc resection of the celiac trunk with distal pancreatectomy and total gastrectomy for the treatment of locally advanced gastric cancer, resection of the celiac axis was proven to be feasible and applied thereafter by several surgeons in patients with tumors of pancreatic body and tail [37,39]. Hirano et al. reported on the largest series of resection of infiltrated celiac axis in 23 patients with carcinoma in pancreatic body and tail with a 5-year survival rate of up to 42% [31]. Other reports, although with smaller patients groups also indicated that extended DP could result in prolonged survival [30].

To date it seems worthwhile to apply extended DP in patients with carcinomas in pancreatic body and tail. However, given the fact that not more than 300 cases of extended DP were reported on until now, more control studies with larger series are critical to draw a more convincing conclusion (Table 3).

## Central pancreatectomy

Central pancreatectomy (CP), also known as segmental, middle or medial pancreatectomy, has been proposed as an alternative approach in patients with small, benign or low-grade malignant tumors such as endocrine and cystic neoplasms located in the neck of the pancreas. The rationale for CP is to remove the neoplasm with preservation of the functional parenchyma, and thereby avoiding a major resection such as PD or DP. This method reduces the risk of diabetes and exocrine insufficiency, and maintains the upper digestive and biliary anatomy [40,41].

Since extended tumor resection does not necessarily result in increased long-term survival, although safely performed, secondary parameters such as patient's quality of life may become more and more important. In this context, local (and complete) tumor resection with CP might result in the highest level of postoperative quality of life, if the operation can be performed without increasing the risk of perioperative complications.

Since the first CP was performed in 1984 in a patient with a pancreatic insulinoma [40], approximately another 200 cases of CP have been reported since then for the treatment of benign or low-grade malignant exocrine and endocrine neoplasms such as islet cell carcinoma, vipoma, mucinous cystadenomas, cystadenocarcinoma, cystic papillary tumors, intraductal papillary mucinous neoplasms, and adenocarcinoma in situ [42-45].

Recently, Adham et al. reported on 50 cases of CP associated with a perioperative morbidity of 36% with no patient loss. Interestingly, none of his patients developed de-novo diabetes. The actuarial 5-year survival and pancreatic remnant survival rates were 98% and 95% respectively[1].

Crippa et al. reported on 100 patients requiring CP, which is the largest series in literature up to date. The most common indications were neuroendocrine neoplasms (33%) and serous cystadenoma (27%). When compared to patients in whom extended DP was performed, no differences were observed in overall morbidity, abdominal complications, and pancreatic fistula rate (17% in CP vs. 13% in extended DP). The mean hospital stay was however longer for CP patients (p = 0.005). After a median follow-up of 54 months, the incidence of de-novo onset of endocrine and exocrine insufficiency was significantly higher in the group of patients with extended DP (4% vs. 38%, p = 0.0001, and 5% vs. 15.6%, p = 0.039, respectively) [42].

Conclusively, CP might be a safe and effective treatment option for small and/or low-grade malignant neoplasms as well as for pancreatic metastases located in the mid-portion of the pancreas, since it is associated with a low risk of exocrine and endocrine insufficiency. However, it is questionable whether this technique will also be applicable in patients suffering from ductal adenocarcinoma, because this tumor entity shows different and more aggressive tumor biology.

## Total pancreatectomy

Total pancreatectomy (TP) for pancreatic cancer was reported by Billroth as early as 1884, and later by Rockey in 1943 [46]. It was not recommended and even abandoned by most surgeons for a long time because of high peri- and postoperative morbidity and mortality. The incidence of postoperative diabetes control problems ranged from 15 to 75%, and according to several studies was the cause of death in the long-term in nearly half of all patients [47].

In the last decades, however, TP has become an adequate treatment option [48,49], since remarkable improvements in both surgery and postoperative management of the apancreatic patient with successful management of endocrine and exocrine insufficiency were achieved. The rationale for total pancreatectomy comes from i) the argument that total pancreatectomy is a better oncologic procedure with wider lymphadenectomy and tissue resection, ii) the tendency that pancreatic cancer is multicentric, and iii) the absence of the pancreaticoenterostomy presenting a less risky procedure in terms of postoperative complications [50].

However, most large retrospective series have not shown any long-term survival benefit. The overall mortality rate was about 9% and morbidity about 45% [3,51-58] (Table 4). Nevertheless, a recently published prospective study on 147 patients showed that both postoperative morbidity and mortality can be kept with low incidence (24% and 4.8% respectively), and that global health status of TP-treated patients was comparable to that of PD patients after a median follow-up of 23 months, although all patients required insulin and exocrine pancreatic enzyme replacements [49]. In the same line of evidence, Schmidt and his colleagues conducted an analysis of 1.579 patients who underwent PD or TP for pancreatic carcinoma. Of these, 33 patients had conversion to TP for isolated neck margin involvement to achieve R0 resection. Interestingly, these patients experienced a greater median survival (18 vs. 10 months; *p *= .004) than R0-resected PD patients [52]. Similarly, Billings et al. reported a relatively high 5-year survival of 34% with a median survival of 24 months in patients with malignant tumors treated by TP [54].

**Table 4:** Reports on total pancreatectomy (TP).

Author (Year of publication)	Number of patients	Mortality [%]	Morbidity [%]	Survival
Muller[49] (2007)	147	4.8	24	21.9 months (median); 1-YS: 64.3%; 5-YS: 36.6%
Schmidt[52] (2007)	33	6	36	18 months*
Jin[55] (2007)	21	23.8	57.1	9.2 months (median)
Billings[54] (2005)	99	5	32	24 months (median); 5-YS: 34%
Wagner[55] (2001)	22	4.5	59	3-YS: 11%; 5-YS: 0%
Bendix [56](2001)	6	0	NA	alive 5–56 months (papillary mucinous tumor)
Ihse[51] (1996)	89	27	52	7 months (median); 5-YS: 4.5%
Swope[57](1994)	47	8	39	526 days *
Launois[58] (1993)	47	15 (before1981) 0 (after 1981)	NA	14.4 months (mean); 1-YS: 42.4% 2-Ys: 25.6% 3-YS: 11.9% 5-YS: 8%

**Overall**	**511**	**8.8**	**44.9**	

* statistically significantly different compared to standard PD

NA: not available; YS: year survival

Undoubtedly, a TP is absolutely reasonable in order to achieve an R0 resection in case of tumor-infiltrated margins after PD or distal resection. However, presently there are not enough studies in favor of TP, and this perhaps requires further prospective investigations. Therefore, most surgeons nowadays do not recommend TP as a routine procedure for the management of pancreatic cancer, although postoperative diabetes control is feasible and low perioperative mortality can be achieved in high-volume centers [3,48].

A further argument in favor of TP is tumor multicentricity, but this still needs to be clearly elucidated, since in some studies tumor dissemination was reported to occur in more than 30% of cases (in 1960s), which is contrary to other studies with only 0% and 6% (in 1990s) [58-61]. Overall, TP is currently only indicated in locally extended pancreatic tumors to be resected with tumor-free surgical margins.

## Laparoscopic pancreatectomy

Laparoscopic techniques can be used for diagnosis, staging and therapeutic procedures)[62]. The first PD performed laparoscopically in 1993 by Gagner et al. was a landmark in spite of their own comment, that the laparoscopic Whipple procedure might not improve the postoperative outcome or shorten the postoperative recovery period, though technically feasible [63,64].

Even nowadays laparoscopic pancreatectomy has not been universally accepted yet. On the contrary, it is criticized by most surgeons and thus rarely performed because of the technical difficulties involved, such as long operating time, increased hospitalization due to delayed gastric emptying, and most importantly due to no improved survival [65].

However, Palanivelu et al. recently conducted a retrospective study on 42 selected patients who underwent laparoscopic PD. Five-year survival rates for all patients with malignancy, ampullary adenocarcinoma, pancreatic cystadenocarcinoma, pancreatic head adenocarcinoma and common bile duct adenocarcinoma were 32%, 30.7%, 33.3%, 19.1%, and 50% respectively. The mortality was nil. They concluded that laparoscopic PD can be performed safely and that good results in carefully selected patients with localized malignant lesions, irrespective of histopathology, can be achieved with this approach [65]. Pugliese et al. also reported on 19 patients with pancreatic neoplasm of the head who were approached by the minimally invasive technique, of which at least 6 of them received laparotomy due to bleeding and difficulties in parenchyma dissection [66].

In addition, Gumbs et al. operated 22 patients with noninvasive intraductal papillary mucinous neoplasms (9 by laparoscopy and 13 by open surgery). PD was performed in 8 patients (3 by laparoscopy and 5 by open). Two patients underwent laparoscopic total pancreatectomy. One patient received open surgery (11%) due to difficulties in reconstructing the biliary anastomosis. The overall complication rates were 56% for the laparoscopic group and 85% for the open group. The mean survival between both groups was not significantly different (20 vs. 37 months, p > 0.05)[67]. With regard to endocrine tumors, the same authors reported on 31 patients of whom 13 (42%) were operated using open techniques and 18 (58%) laparoscopically. Only one of these patients received a conversion (6%). In the laparoscopic group eight (47%) tumors were malignant compared to six (43%) in the open group. The overall actuarial survival rates were both about 90% at 5 years, and operations performed laparoscopically were performed faster than open surgery [68].

Fernandez-Cruz et al. reported on 49 consecutive patients with neuroendocrine tumors who underwent laparoscopic pancreatic surgery. The benefits of minimally invasive surgery were manifest during the short hospital stay (spleen preserving DP: 5.9 days; spleen resection DP: 7.5 days; laparoscopic enucleation: 5.5 days) and acceptable pancreas-related complications (22–42.8%) in high-risk patients [69].

Nevertheless, studies on this procedure contain only small numbers of patients and are usually performed without corresponding control groups. Therefore, laparoscopic PD in patients with malignant tumor still remains controversial, though some prospective studies are already in progress by experienced surgeons in several large centers. Although these studies are not large, they have however proved the feasibility of laparoscopic surgery in pancreatic tumor disease, at least with no poorer results than with open PD.

Laparoscopic DP on the other hand (with or without preserving the spleen) is technically easier and more widely accepted [70]. Nevertheless, it has as yet not become as popular as other laparoscopic surgeries. Demonstrating the feasibility of this technique, Palanivelu et al. reported on 22 patients who underwent laparoscopic DP with (n = 15) or without (n = 7) splenectomy. All patients were started on a liquid diet on the first postoperative day, and median hospital stay was 4 days. Only one patient developed a pancreatic fistula that was managed conservatively. There was no recurrence noticed during an average follow-up of 4.6 years [70].

Sa Cunha et al. retrospectively studied sixty patients with presumed pancreatic neoplasms, of which 57 (95%) were benign and 3 (5%) malignant. Successful laparoscopic procedures included 20 DP with spleen preservation, 5 DP with splenectomy, 16 enucleations, 5 CP, 1 PD, and 1 TP. Postoperative death occurred in one patient (1.6%) due to mesenteric ischemia after tumor enucleation. The overall postoperative complication rate was 36%, including a 13% rate of clinically obvious pancreatic fistulae. In successful laparoscopic operations the mean postoperative hospital stay was 12.7 days[71].

All these series proved that laparoscopic DP may benefit patients, since this procedure was associated with reduced postoperative pain, shorter hospital stay, faster recovery and return to normal activity, better cosmetic appearances, and most of all an improved long-term survival. Additionally, laparoscopy can be reliably utilized for biopsies, thus reducing or avoiding unnecessary laparotomy, especially in patients with autoimmune pancreatitis [2], and may also be useful during preoperative staging of pancreatic tumors by avoiding unnecessary explorative laparotomy [72].

Consequently, laparoscopic DP with or without spleen preservation has been considered as a safe procedure. For malignant pancreatic tumors, laparoscopic DP should however only be performed in selected patients, whereas laparoscopic PD should be reserved only for highly skilled laparoscopic surgeons. Validation of these advanced procedures by clinical trials is still required [2].

## Conclusion

Since no other appropriate treatment modalities are available to increase the outcome of patients with malignant pancreas tumors, only the extension of so far standardized surgical strategies seems to be mandatory. This is another typical example of primitive surgeons' motive to achieve better results through expansion of resection by the scalpel in their hands.

PD with standard lymphadenectomy with vascular resection is still the optimal surgical procedure for carcinomas in the head of pancreas. For those patients with small or low-grade malignant neoplasms as well as small pancreatic metastases located in the mid-portion of pancreas, CP is emerging as a safe and effective option with a low risk for development of exocrine and/or endocrine insufficiency. TP is not as risky as it was years before and can nowadays safely be performed, but its indication is limited to locally extended tumors that cannot be removed by PD or DP with tumor-free surgical margins. Consequently, TP has not been adopted as a routine consideration by most surgeons, whereas aggressive surgical features are warranted in case of advanced distal pancreatic tumors, provided safe and experienced surgery is available to achieve complete (R0) tumor removal.

With the development of modern instruments, laparoscopic operations could be performed more successfully, even in malignant pancreatic diseases, thereby representing the prospective issue in pancreatic surgery.

## Competing interests

The authors declare that they have no competing interests.

## Authors' contributions

MG gave substantial contributions to conception and design, analysis and interpretation of data, has been involved in drafting and revising the manuscript, has given final approval of the version to be published. BS gave substantial contributions to conception and design, analysis and interpretation of data, has been involved in drafting and revising the manuscript, has given final approval of the version to be published. FL gave substantial contributions to acquisition and analysis of data, has given final approval of the version to be published. XS gave substantial contributions to acquisition and analysis of data, has given final approval of the version to be published. MB gave substantial contributions to acquisition and analysis of data, has been involved in drafting and revising the manuscript, has given final approval of the version to be published. DJ gave substantial contributions to acquisition and analysis of data, has been involved in drafting and revising the manuscript, has given final approval of the version to be published. UN has been involved in drafting and revising the manuscript, has given final approval of the version to be published. PN gave substantial contributions to conception and design, has given final approval of the version to be published.
